# Takotsubo Cardiomyopathy Associated With Influenza A and a Ground-Level Fall

**DOI:** 10.7759/cureus.65337

**Published:** 2024-07-25

**Authors:** Ashwin Jagadish, Shahnawaz N Notta, Colin McGuire, Lalith Namburu, Shobha Hiremagalur

**Affiliations:** 1 Internal Medicine, East Tennessee State University James H. Quillen College of Medicine, Johnson City, USA; 2 Cardiology, East Tennessee State University James H. Quillen College of Medicine, Johnson City, USA; 3 Cardiology, Ballad Health CVA Heart Institute, Johnson City, USA

**Keywords:** cardiology, ground level fall, influenza, takotsubo cardiomyopathy, stress cardiomyopathy

## Abstract

Our case involves a 92-year-old female who presented to the emergency department due to a ground-level fall and difficulty breathing. She was found to have influenza A, elevated troponin, and decreased left ventricular ejection fraction. However, cardiac catheterization did not reveal any coronary artery stenosis, supporting a diagnosis of takotsubo cardiomyopathy (TC). The patient’s ejection fraction was normal after nine months. This case highlights the importance of considering TC in elderly female patients who have reduced ejection fraction and elevated troponin in the setting of infection and a recent fall.

## Introduction

Takotsubo cardiomyopathy (TC) was first reported in 1991 in Japan [1]. Alternative names for the condition include broken heart syndrome, stress cardiomyopathy, and Gebrochenes-Herz syndrome [2]. TC can be associated with accidents, trauma, illnesses, abuse, stimulant substances, financial troubles, or the death of loved ones [2]. Physical triggers may be a more prevalent source of TC inducement than emotional triggers; additionally, the absence of a trigger should not result in excluding a diagnosis of TC [3].

TC may occur in individuals of any age but is most frequently seen in those between the ages of 60 and 70 [4]. Roughly 80-90% of affected individuals are female [4]. TC is present in approximately 2-3% of all cases of acute coronary syndrome (ACS) [4]. It is seen in 5-6% of female individuals with ACS [4]. Individuals who develop TC due to physical stressors may have a higher mortality rate than those who develop the condition after an emotional stressor [5].

Developing TC in association with influenza is uncommon, with a 2022 systematic review identifying only 10 case reports within the literature [6]. Our case involves an elderly female individual who developed TC in association with multiple factors: a ground-level fall and influenza A.

## Case presentation

**Figure 1:**
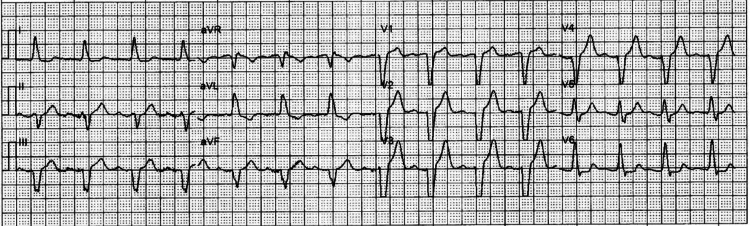
ECG obtained by emergency medical services The electrocardiogram (ECG) demonstrates a left bundle branch block.

A 92-year-old female with a history of pacemaker placement approximately 25 years ago presented to the emergency department due to a ground-level fall. She was reportedly ambulating within her house when she suddenly fell down and appeared to hit her head on some stairs. The fall was not witnessed, but her son quickly responded after hearing her call for help. After the fall, the patient was having difficulty breathing and was gasping for air, so emergency medical services (EMS) was notified. While under the care of EMS, her oxygen saturation was between 70% and 80%, so she was placed on mid-flow oxygen at 10 L. Subsequently, her oxygen saturation improved to slightly above 90%. The patient was noted to have had a recent exposure to influenza and also a dry cough for a few days. An electrocardiogram (ECG) conducted by the EMS team revealed a left bundle branch block (Figure 1).

In the emergency department, she had a blood pressure of 134/72 mmHg, pulse of 76 beats per minute, temperature of 98.3°F (36.8°C), respiratory rate of 19 breaths per minute, and oxygen saturation of 93%. Her laboratory evaluation can be seen in Table 1. On physical examination, she was found to have a scalp contusion. On auscultation, she was noted to have decreased air exchange, rhonchi, and crackles bilaterally. Her cardiovascular examination revealed a regular rate and rhythm.

**Table 1:** Laboratory studies obtained in the emergency department The table indicates abnormal laboratory values identified on presentation to the emergency department.

Laboratory test	Value	Reference range
White blood cell	17.4 K/µL	3.5-10.5 K/µL
Sodium	133 mmol/L	137-145 mmol/L
Creatinine	1.22 mg/dL	0.52-1.04 mg/dL
Blood urea nitrogen	23.9 mg/dL	7.0-17.0 mg/dL
Lactate	2.5 mmol/L	0.5-2.0 mmol/L
Troponin I	0.50 ng/mL	0.00-0.03 ng/mL

**Figure 2:**
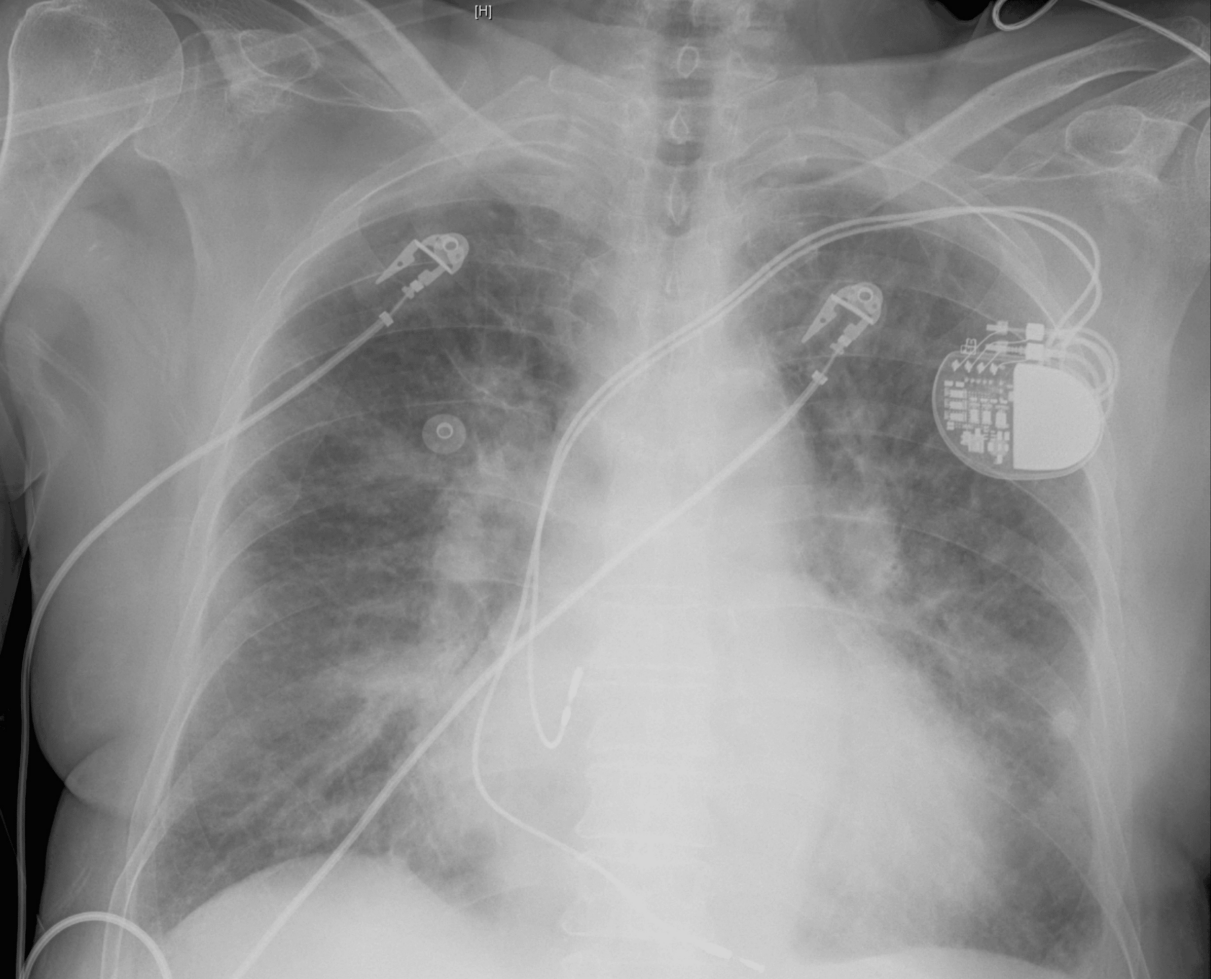
Chest radiograph The radiograph is suspicious for congestive heart failure with pulmonary edema versus multifocal pneumonia.

**Figure 3:**
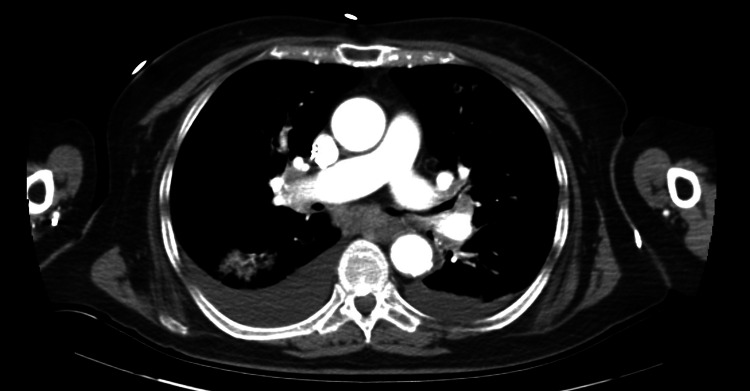
CT of the chest, abdomen, and pelvis Computed tomography (CT) of the chest, abdomen, and pelvis revealed small to moderate right and small left pleural effusions, pulmonary edema, multifocal pneumonia, and atherosclerosis.

Initial chest x-ray was concerned for congestive heart failure with pulmonary edema versus multifocal pneumonia (Figure 2). Computed tomography (CT) of the chest, abdomen, and pelvis revealed small to moderate right and small left pleural effusions, pulmonary edema, multifocal pneumonia, and atherosclerosis (Figure 3). CT of the head did not reveal any acute processes. CT of the cervical, thoracic, and lumbar spine did not reveal any acute fractures. She was started on 1 g of intravenous ceftriaxone every 24 hours and 100 mg of doxycycline every 12 hours. The respiratory viral panel was positive for influenza A, so 30 mg of oseltamivir twice daily was also initiated.

**Figure 4:**
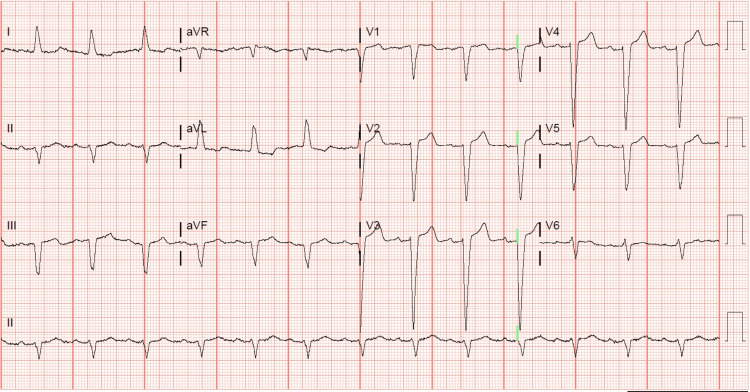
ECG obtained in the emergency department The electrocardiogram (ECG) demonstrates a left bundle branch block.

**Figure 5:**
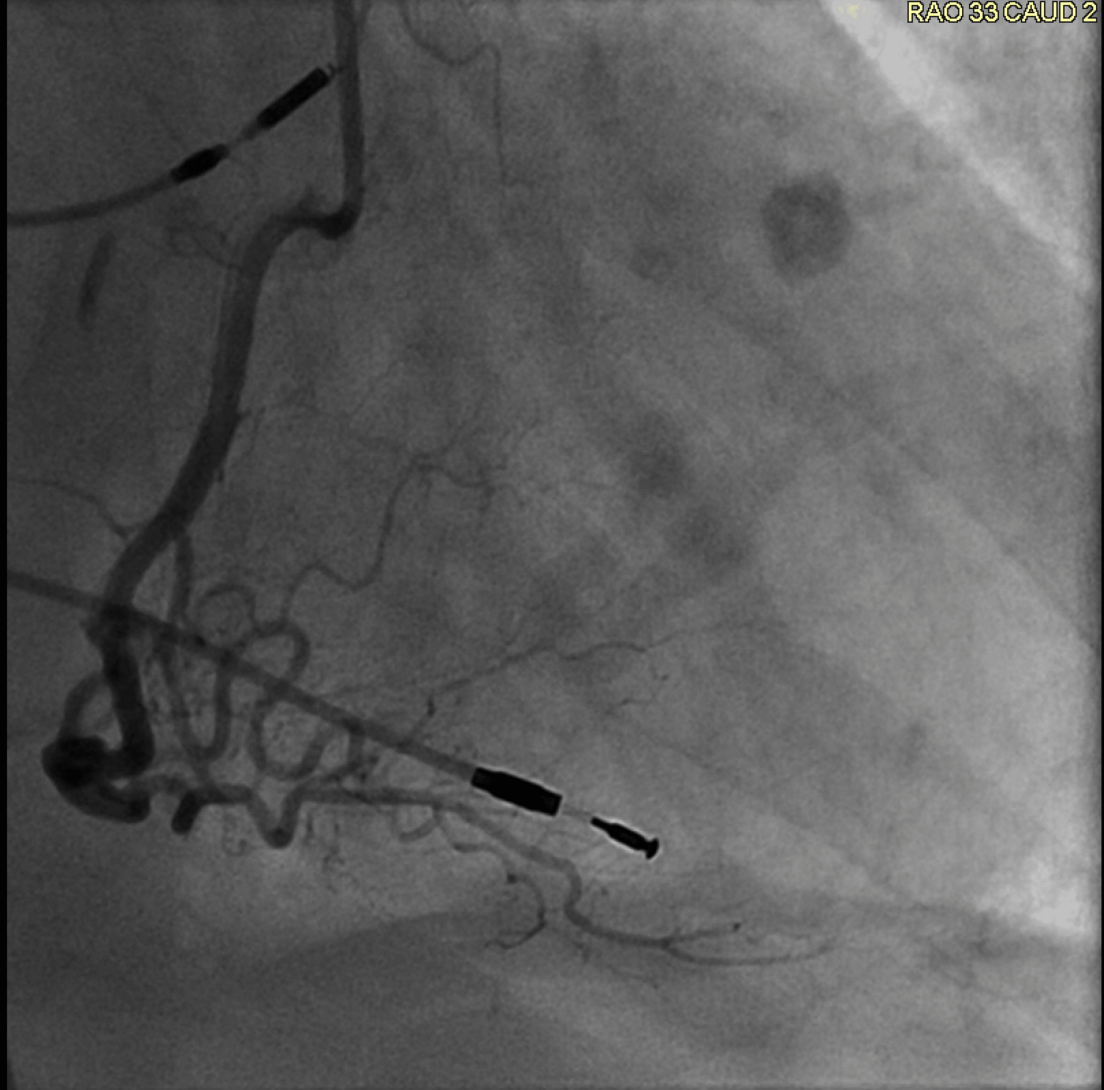
Right coronary angiogram The right coronary angiogram does not reveal any stenosis.

**Figure 6:**
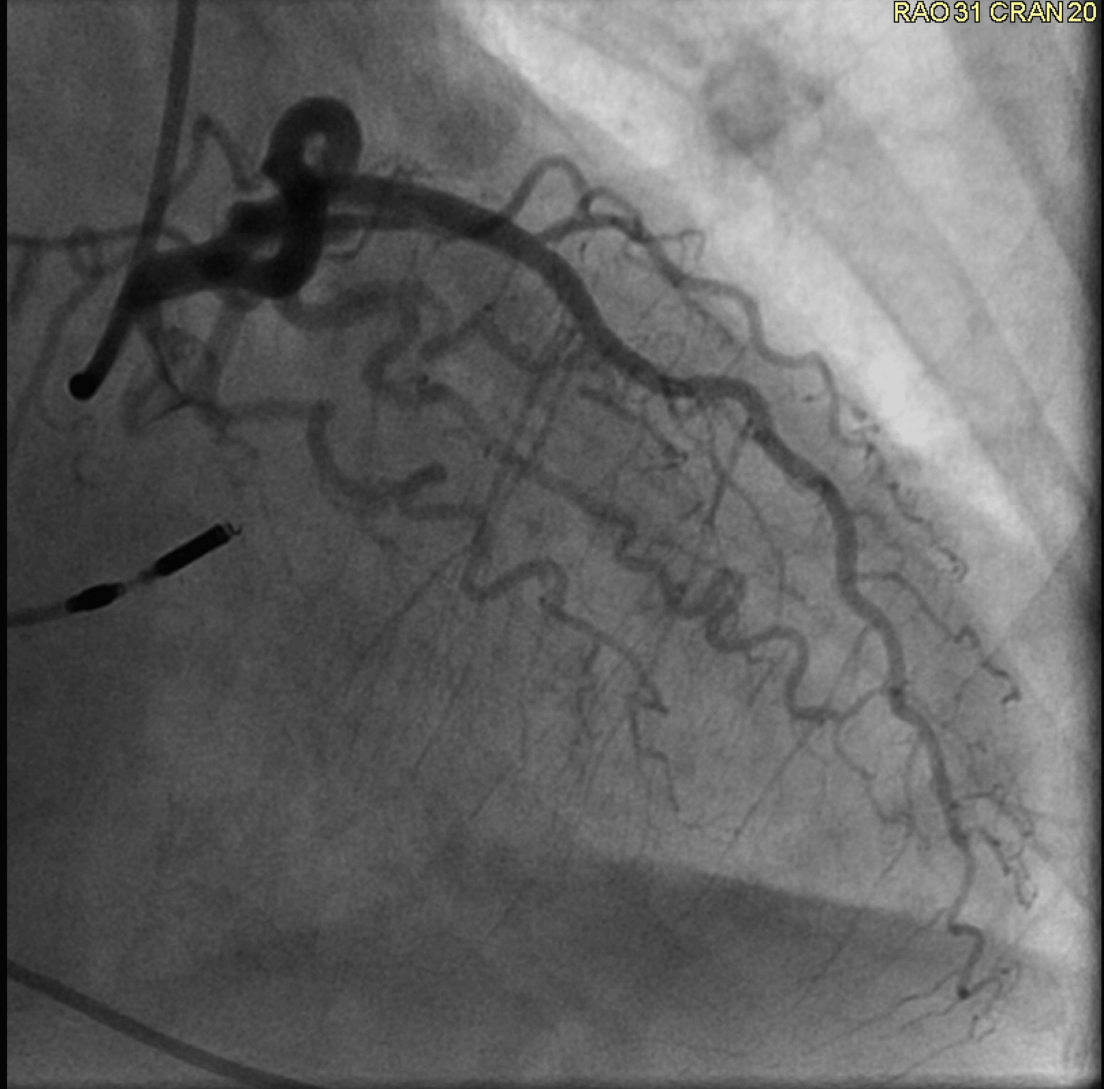
Left coronary angiogram The left coronary angiogram does not reveal any coronary artery stenosis.

**Figure 7:**
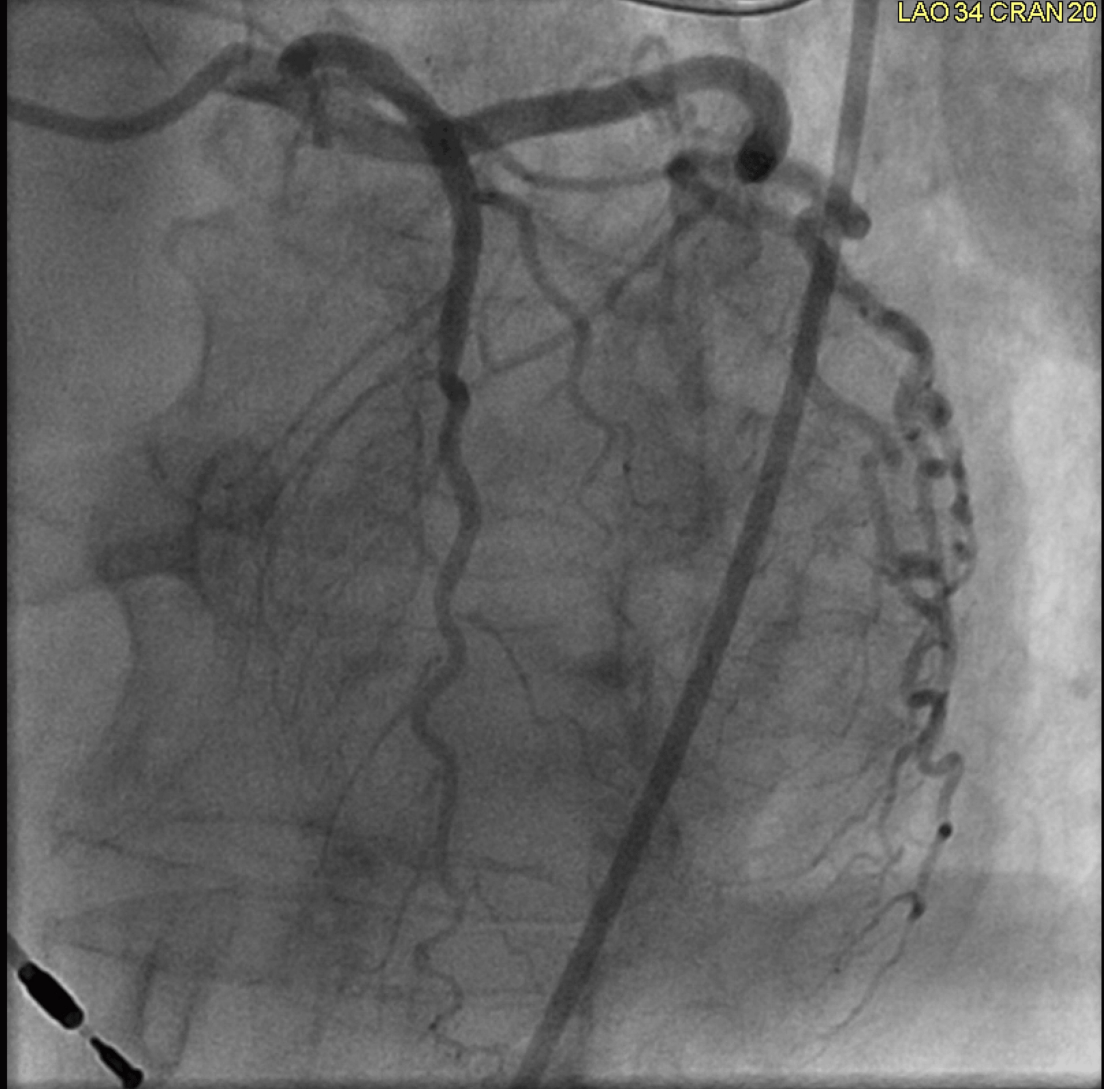
Left coronary angiogram The left coronary angiogram does not reveal any coronary artery stenosis.

**Figure 8:**
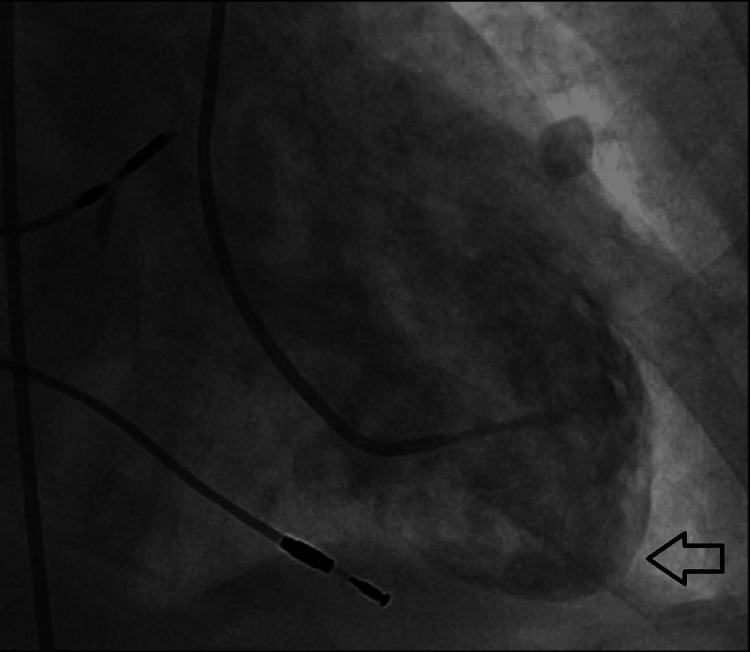
Left ventriculogram in systole The left ventricular apex (indicated by the arrow) is akinetic in systole.

**Figure 9:**
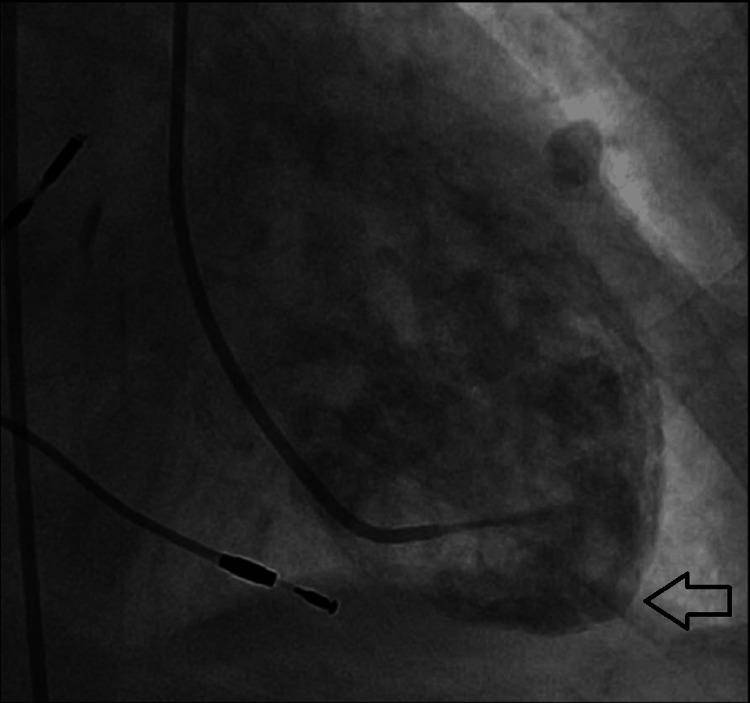
Left ventriculogram in diastole The left ventricular apex (indicated by the arrow) as seen in diastole.

Her ECG also demonstrated the new left bundle branch block, which was initially discovered by the EMS team (Figure 4). A transthoracic echocardiogram revealed a left ventricular ejection fraction (LVEF) of 20-25%. In addition, it revealed akinesis of the mid and distal anterior septum, apical anterior segment, apical inferior segments, and apex. Hypokinesis of anterior wall, entire lateral wall, inferior wall, basal anteroseptal segment, mid inferoseptal segment, and basal inferoseptal segment was noted. There was also some mild mitral valve regurgitation and moderate tricuspid regurgitation. Her most recent echocardiogram, completed two months prior, indicated a normal LVEF of 60-65%. Subsequent cardiac catheterization revealed an LVEF of 20-25%. Right and left coronary angiograms did not reveal any significant arterial stenosis (Figures 5-7). In addition, the left ventriculogram in systole revealed the apex to be akinetic (Figure 8). The left ventriculogram in the diastole is seen in Figure 9. Thus, the patient was diagnosed with TC. She was subsequently started on 75 mg of clopidogrel once daily, 25 mg of losartan once daily, 100 mg of metoprolol succinate once daily, and 5 mg of rosuvastatin once daily.

The patient ultimately completed a five-day course of both ceftriaxone and doxycycline, in addition to a seven-day course of oseltamivir. She was subsequently discharged to a skilled nursing facility with instructions to continue the newly started cardiac medications and to follow up as an outpatient with her cardiologist. The patient declined a LifeVest® wearable defibrillator. A follow-up echocardiogram completed nine months later indicated a normal LVEF of 55-60%. Mild mitral and tricuspid regurgitation were present.

## Discussion

The signs of TC are similar to those of ACS [2]. Patients can present with chest pain, shortness of breath, syncope, or even pulmonary edema [7]. ECG can demonstrate ST-segment elevation, T-wave abnormalities, and prolonged QT interval [4]. Laboratory results can show increased levels of troponin, C-reactive protein, and brain natriuretic peptide [4]. Echocardiography findings can include transient left ventricular wall movement anomalies [4] and decreased LVEF, sometimes as low as 20% [7]. However, in the case of TC, coronary angiography should not reveal any obstructions [4]. Although, apical ballooning can be present [4]. 

Since TC presents similarly to ACS, the initial treatment regimen is often similar [3]. Since abnormalities can be present in troponin level and ECG findings, neither test can be used to definitively rule out ACS [3]. As a result, coronary angiography may be needed to make a formal diagnosis [3].

TC was previously thought to be a benign condition; however, there are adverse outcomes associated with the condition [3]. Compared to individuals with ACS, those with TC are more likely to have a psychiatric or neurological condition [3]. Up to 20% of patients with TC can have a severe complication while hospitalized [3]. The rate of death per patient-year can be as high as 5.6% [3]. Treating patients with angiotensin receptor blockers (ARB) or angiotensin-converting enzyme (ACE) inhibitors can have survival benefits in patients with TC [3]. 

While the exact mechanism behind TC has not been identified, there is evidence suggesting that catecholamines are involved [8]. Catecholamine levels can be higher in individuals with TC than in those with myocardial infarctions [8]. The duration of catecholamine elevation can also be longer in these individuals [8]. There is evidence of elevated norepinephrine levels within the coronary sinus of individuals with TC, alluding to increased local release of catecholamines [8]. There may be a “stunning” phenomenon associated with large amounts of catecholamines [8]. In support of the catecholamine theory is the knowledge that the administration of catecholamines, such as epinephrine or norepinephrine, can directly lead to TC [8].

## Conclusions

Our case involves an elderly female who developed TC in the setting of a ground-level fall and influenza A. TC can present in individuals of all ages but primarily impacts women in the sixth decade of life. TC is associated with both physical and emotional triggers, and increased mortality is thought to be associated with physical triggers. Patients often present similarly to those with ACS, so coronary angiography may be necessary to definitively diagnose TC. While initially thought to be benign, TC is actually associated with adverse outcomes, such as increased risk of inpatient complications and death.
